# Origin and Diversification of Major Clades in Parmelioid Lichens (Parmeliaceae, Ascomycota) during the Paleogene Inferred by Bayesian Analysis

**DOI:** 10.1371/journal.pone.0028161

**Published:** 2011-12-08

**Authors:** Guillermo Amo de Paz, Paloma Cubas, Pradeep K. Divakar, H. Thorsten Lumbsch, Ana Crespo

**Affiliations:** 1 Departamento de Biología Vegetal II, Facultad de Farmacia, Universidad Complutense, Madrid, Spain; 2 Department of Botany, The Field Museum, Chicago, Illinois, United States of America; American Museum of Natural History, United States of America

## Abstract

There is a long-standing debate on the extent of vicariance and long-distance dispersal events to explain the current distribution of organisms, especially in those with small diaspores potentially prone to long-distance dispersal. Age estimates of clades play a crucial role in evaluating the impact of these processes. The aim of this study is to understand the evolutionary history of the largest clade of macrolichens, the parmelioid lichens (Parmeliaceae, Lecanoromycetes, Ascomycota) by dating the origin of the group and its major lineages. They have a worldwide distribution with centers of distribution in the Neo- and Paleotropics, and semi-arid subtropical regions of the Southern Hemisphere. Phylogenetic analyses were performed using DNA sequences of nuLSU and mtSSU rDNA, and the protein-coding *RPB1* gene. The three DNA regions had different evolutionary rates: *RPB1* gave a rate two to four times higher than nuLSU and mtSSU. Divergence times of the major clades were estimated with partitioned BEAST analyses allowing different rates for each DNA region and using a relaxed clock model. Three calibrations points were used to date the tree: an inferred age at the stem of Lecanoromycetes, and two dated fossils: *Parmelia* in the parmelioid group, and *Alectoria*. Palaeoclimatic conditions and the palaeogeological area cladogram were compared to the dated phylogeny of parmelioid. The parmelioid group diversified around the K/T boundary, and the major clades diverged during the Eocene and Oligocene. The radiation of the genera occurred through globally changing climatic condition of the early Oligocene, Miocene and early Pliocene. The estimated divergence times are consistent with long-distance dispersal events being the major factor to explain the biogeographical distribution patterns of Southern Hemisphere parmelioids, especially for Africa-Australia disjunctions, because the sequential break-up of Gondwana started much earlier than the origin of these clades. However, our data cannot reject vicariance to explain South America-Australia disjunctions.

## Introduction

In traditional, morphology-based concepts, lichenized fungi often have wide distributions spanning over several continents with a number of species being cosmopolitan. This has led to a widespread notion that the distribution of these fungi is primarily shaped by ecological conditions rather than explained by historical events. In contrast, a few authors have invoked plate tectonics and emphasized vicariance to explain distribution patterns of lichens, especially for species occurring in the Southern Hemisphere [1]–[6]. However, within the last decade, molecular data have helped to revolutionize the species delimitation of lichenized fungi and demonstrated that morphology-based concepts largely underestimate the diversity of lichens [7]–[16]. As a result of these studies, it became clear that – although the number of widely distributed species in lichenized fungi is generally higher than in plants or animals – lichens have more restricted distribution areas than previously assumed. For example, supposedly cosmopolitan species in Parmeliaceae and Physciaceae were found to represent distinct lineages in different continents [11], [13]. Further, recent progress in our knowledge of the phylogeny of some clades of lichenized fungi revealed the presence of clades at generic rank that originated and diversified in the Southern Hemisphere [17]–[21]. Hence, we have turned our attention to address the issue of the extent of vicariance and long-distance dispersal to understand the current distribution of lichenized fungi anew using molecular phylogenies.

For this purpose a dated phylogeny with the estimated ages of origin and diversification of the parmelioid group is required. A main problem for building dated phylogenies in fungi is the poor fossil record. While our understanding of the divergence time of angiosperms is well established [22]–[25],. time estimates for fungi were long disputed based on uncertainties in the interpretation of the few known fossils [26]. Consequently, published dating estimates ranged from 660 million to 2.15 billion years ago for the origin of Fungi and from 390 million to 1.5 billion years for the split of the two crown groups of fungi, Ascomycota and Basidiomycota [27]–[31]. Re-examination of the morphology of the fossils and re-evaluation of the published dating studies, however, suggested more consistent results with the origin of the Fungi dating back to between 760 million and 1.06 billion years, and the split of Ascomycota and Basidiomycota for about 500–650 Ma [26], [32]. This suggests that terrestrial fungi evolved and diversified more or less simultaneously with the evolution of land plants.

Given that the phylogeny of parmelioid lichens is well resolved [18] and the recent advantages in our understanding of the timing of major events in the evolution of fungi, we feel confident that the times of main nodes differentiation can be estimated. Moreover, this dating approach can be applied to address the issue of the extent of vicariance on distribution patterns in lichens and use parmelioids as an example.

**Figure 1:**
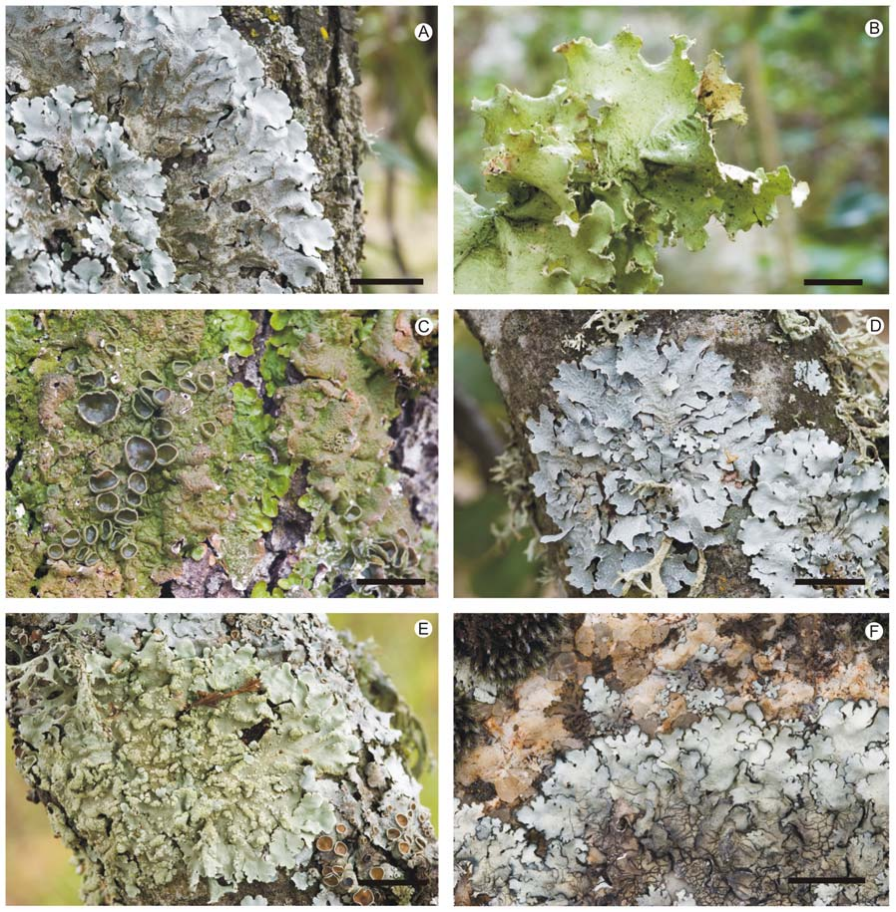
Selected examples of different genera and species of parmelioid lichens. A. *Parmelina tiliacea*, one of the most common lichens in the Mediterranean basin. B. *Parmotrema hypoleucinum*, endemic of the southwestern Mediterranean Region, occurring in warm and humid areas. C. *Melanelixia glabra* occurs from southwestern Europe to eastern Russia. D. *Parmelia sulcata*, a common species from cold to temperate regions of both Hemispheres. E. *Flavoparmelia soredians* occurs in warm and humid areas of temperate regions of both Hemispheres. F. *Xanthoparmelia conspersa*, one of the most widespread species of macrolichens growing on acid rocks in temperate areas of both Hemipheres, excluding Australia and South Africa. The distribution areas after Nimis [105]. All photographs were taken in the field. Scale = 1 cm.

The family Parmeliaceae is widely distributed throughout the world from polar to tropical regions and is one of the largest families of lichenized Ascomycota [18], [33]–[36]. Most species form foliose or fruticose thalli, but some also have subcrustose, umbilicate, peltate thalli, and even lichenicolous fungi were found to belong here [18], [35], [37]. The family includes about 2500 species classified in 84 genera and is characterized by cup-shaped apothecia with cupulate exciple, *Lecanora*-type asci, often with hyaline ascospores [35]. Within the family, six strongly supported major monophyletic groups can be distinguished [35], which are: alectorioid, cetrarioid, hypogymnioid, letharioid, parmelioid and psiloparmelioid. By far, the largest of these groups is the parmelioid group with about 1500 species [38], [39] classified in 27 accepted genera [18]. Phenotypically, the parmelioid lichens are characterized by having mostly foliose thalli with thread-like rhizines on the lower surface, cup-shaped apothecia on the thallus upper surface and *Lecanora*-type asci with hyaline ascospores (Fig. 1). Within the parmelioid lichens, eight major monophyletic clades can be distinguished, which are *Cetrelia-*, *Hypotrachyna-*, *Melanohalea-*, *Parmelia-*, *Parmelina-*, *Parmeliopsis-*, *Parmotrema-* and *Xanthoparmelia*-groups [18]. During the last decades, phylogenetic studies based on DNA sequence data have greatly advanced our understanding of the evolution of the family including the phylogenetic relationships among major clades [18], [34]–[35], [40]–[46], phenotypical evolution [35], [45], [47], disparity in substitution rates among clades [48], and the geographic origin of certain clades [17], [19]. However, so far none of these have aimed at dating major cladogenesis events in Parmeliaceae. Besides the issues discussed above, this is probably also due to the poor fossil record of Parmeliaceae. Only very few fossils have been recorded of Parmeliaceae and the known ones are all preserved in amber. An *Alectoria* species was described from Baltic amber (35–40 Ma) [49]. Two fossil species of *Parmelia* were described from Dominican amber (15–45 Ma) [50] and two specimens of *Anzia* have been found from European amber (35–40 Ma) [51].

The aims of this study are 1) to put a time-scale on the phylogenetic tree of parmelioid lichens and thus identify when the main nodes differentiated, employing calibration points from a recent dating estimate of fungi [26] and available fossil reference-points, and 2) to address the impact of vicariance and long-distance dispersal processes in the distribution patterns in parmelioid lichens. Specifically we address the question whether plate tectonics can be invoked to explain distribution areas of species groups or species occurring in the Southern Hemisphere.

## Results

### Data and substitution patterns

For the analyses a dataset of three loci of 225 OTUs was used. The matrix included 1849 unambiguously aligned nucleotide characters, with 599 positions in the *RPB1*gene, 655 positions in the nuLSU and 595 positions in the mtSSU. The number of constant characters was 643. The likelihood value of the ML tree obtained with Garli was lnL = −59525.412. The constrained position of Chaethothyriomycetidae as sister group of Lecanoromycetes is not significantly worse than the unconstrained topology (*p*-SH = 0.444, *c*-ELW = 0.441).

**Figure 2:**
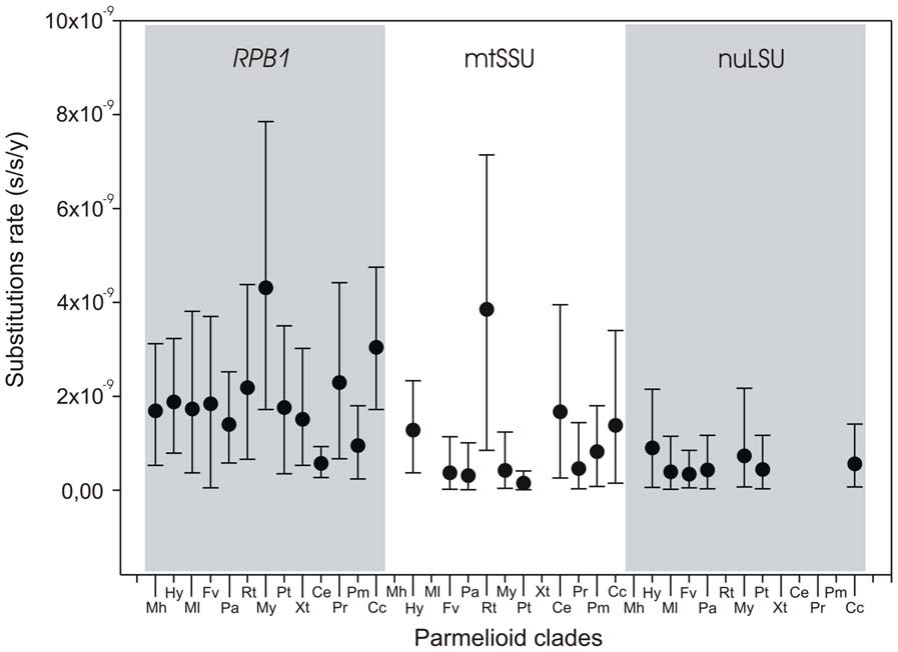
Substitution rates for the three loci (mtSSU, nuLSU, *RPB1*) of the main clades in Parmelioid. Dots represent mean rates and bars cover the 95% highest posterior density (HPD). Units: substitution/site/year. Abbreviations for the Parmelioid clades are listed in Table 1.

The substitution rates of the three loci (nuLSU, mtSSU and RPB1) are shown for each lineage of the parmelioid lichens in Fig. 2 and Table 1. The different lineages have similar range of variation in each gene but there are clear differences in mean rates between the three genes, the rate of *RPB1* being two to four times higher than mtSSU and nuLSU, respectively.

**Table 1:** Substitution rates of the main clades of parmelioid lichens obtained in the independent BEAST analyses for each locus.

	RPB1		mtSSU		nuLSU	
Node MRCA	Rate	Rate 95% HPD	Rate	Rate 95% HPD	Rate	Rate 95% HPD
Lecanoromycetes	2.01	0.37–4.89	0.67	0.01–1.80	0.28	0.02–0.77
Lecanoromycetidae	1.68	0.25–3.75	0.51	0.02–1.31	-	-
Lecanorales	1.65	0.39–3.26	0.84	0.09–1.89	0.71	0.06–1.84
Parmeliaceae (*Protoparmelia* included)	1.85	0.49–3.83	-	-	1.12	0.23–2.20
Parmeliaceae (*Protoparmelia* excluded)	2.57	1.13–4.63	0.69	0.08–1.66	0.70	0.13–1.70
parmelioid group	2.58	0.43–5.01	-	-	-	-
*Melanohalea* (Mh)	1.69	0.53–3.12	-	-	-	-
Hypotrachyna-clade (Hy)	1.88	0.79–3.23	1.28	0.37–2.33	0.90	0.06–2.15
*Melanelixia* (Ml)	1.73	0.37–3.81	-	-	0.39	0.02–1.15
*Flavoparmelia* (Fv)	1.84	0.05–3.70	0.37	0.02–1.14	0.34	0.05–0.85
*Parmelia* (Pa)	1.40	0.58–2.52	0.31	0.01–1.01	0.43	0.03–1.17
*Remototrachyna* (Rt)	2.18	0.66–4.38	3.85	0.85–7.14	-	-
*Myelochroa* (My)	4.31	1.72–7.85	0.42	0.04–1.24	0.73	0.07–2.17
*Punctelia* (Pt)	1.76	0.35–3.50	0.15	0.01–0.41	0.44	0.03–1.17
*Xanthoparmelia* (Xt)	1.51	0.53–3.02	-	-	-	-
*Cetrelia* (Ce)	0.57	0.27–0.93	1.67	0.26–3.95	-	-
*Parmotrema* (Pr)	2.29	0.67–4.42	0.46	0.03–1.44	-	-
*Parmelina* (Pm)	0.95	0.24–1.80	0.82	0.08–1.80	-	-
Canoparmelia crozalsiana-clade (Cc)	3.04	1.72–4.75	1.38	0.15–3.40	0.56	0.07–1.41

Units: substitution/site/year ×10^−9^. HPD: highest posterior density interval.

### Estimating divergence times

**Figure 3:**
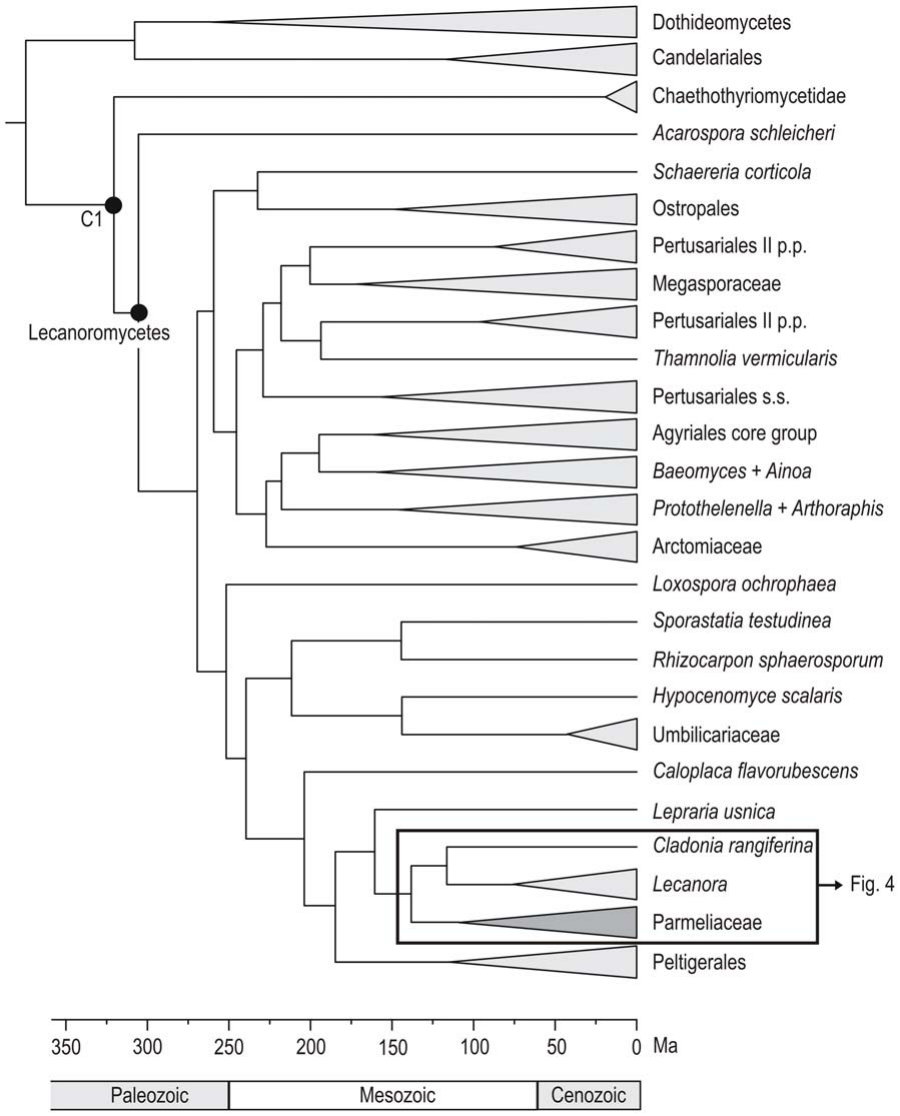
Lecanoromycetes tree indicating the position of the detailed chronogram of Parmeliaceae shown in **Figure 4**. The chronogram was estimated from a partitioned data set of three loci (mtSSU, nuLSU, *RPB1*) using BEAST. The calibration point (C1) was set at the divergence node of Lecanoromycetes and Chaethothyriomycetidae.

**Figure 4:**
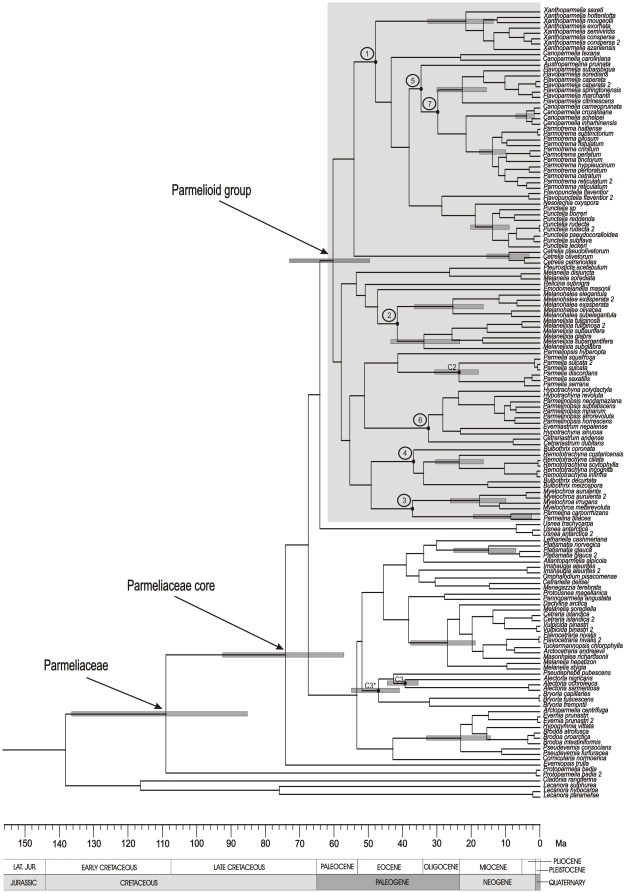
Divergence time chronogram focusing on Parmeliaceae. The chronogram was estimated as described in the legend for figure 3. Two calibration points based on fossil assignation are marked as C2 (crown node of *Parmelia*) and C3 (crown node of *Alectoria*). C3* is the alternative calibration with the *Alectoria* fossil assigned to the alectorioid crown. The Parmelioid group is highlighted by the grey box. Numbers inside circles refer to divergence nodes between the main clades. Grey bars show the 95% highest posterior density intervals (HPD). Detailed ages are given in Table 2.

A chronogram based on the analysis of the combined matrix of three loci is shown in Fig. 3, showing the relationships of Lecanoromycetes and Parmeliaceae (highlighted in dark grey color). The detailed chronogram for Parmeliaceae is depicted in Fig. 4. This analysis used as calibration points the age of the split of Lecanoromycetes-Chaethothyriomycetidae (C1); the age of the fossil (*P. ambra*) assigned to the *Parmelia* s.s. crown node (C2), and the age of the fossil *A. succini* assigned to the *Alectoria* crown (C3). The node ages, 95% highest posterior density intervals (HPD) of ages, substitution rates and 95% HPD of substitution rates for the main clades, divergence points and diversification point of the genera are shown in Table 2.

**Table 2:** Estimated ages and substitution rates of the most recent common ancestors (MRCA) for the main clades obtained with partitioned BEAST analyses using three calibration points.

Period (Epoch) Age	Node MRCA	Age	Height 95% HPD	Rate	Rate 95% HPD
Carboniferous 360–300	Lecanoromycetes	305.53	275.46–327.36	2.64	0.19–6.85
Permian 300–250	Ostropomycetidae	259.33	221.98–293.64	1.31	0.19–3.28
	Lecanoromycetidae	251. 68	211.58–284.79	1.45	0.27–3.17
Jurassic 205–135	Lecanorales	160.65	129.66–192.63	1.40	0.18–2.98
Cretaceous 135–65	Parmeliaceae (*Protoparmelia* included)	108.96	85.52–136.55	2.07	0.52–4.21
	Parmeliaceae core (*Protoparmelia* excluded)	74.17	57.59–92.56	2.92	1.29–4.60
Paleogene (Paleocene) 65–53	Parmelioid group	60.28	49.81–73.55	2.87	0.71–5.78
Paleogene (Eocene) 53–34	Split 1. *Xanthoparmelia* – Parmotrema-clade	47.87	37.28–58.49	1.64	0.24–3.51
	Split 2. *Melanelixia* - *Melanohalea*	41.55	31.25–51.94	1.60	0.27–3.54
	Split 3. *Parmelina* - *Myelochroa*	37.13	24.97–48.65	1.80	0.17–4.71
	Split 4. *Remototrachyna* - *Bulbotrix*	36.78	27.66–45.72	3.81	1.49–6.58
	Split 5. *Austroparmelina*– *Parmotrema, Flavoparmelia, …*	34.60	26.67–42.69	1.41	0.13–3.08
Paleogene (Oligocene) 34–23.5	*Melanelixia*	33.82	23.66–43.86	1.50	0.28–3.26
	Split 6. *Cetrariastrum - Hypotrachyna*-clade p.p.	32.48	22.70–43.70	2.10	0.94–3.61
	Split 7. *Flavoparmelia* - *Parmotrema*	29.73	22.30–36.18	2.78	0.66–6.64
	*Melanohalea*	25.38	16.58–36.73	1.78	0.61–3.53
	*Parmelia*	23.59	18.03–31.31	1.56	0.49–3.10
	*Remototrachyna*	23.54	16.69–30.68	1.50	0.38–2.78
Neogene (Miocene) 23.5–5.3	*Flavoparmelia*	22.61	15.71–30.09	2.25	0.66–4.48
	*Xanthoparmelia*	21.55	13.64–32.74	1.27	0.41–2.35
	*Myelochroa*	17.55	10.00–26.23	3.36	1.45–6.35
	*Punctelia*	13.82	9.17–20.35	1.96	0.30–3.55
	*Parmotrema*	13.80	10.08–17.82	2.64	0.76–5.30
	*Cetrelia*	9.04	3.32–15.62	0.53	0.24–0.89
	*Parmelina*	8.45	2.24–19.49	0.68	0.22–1.25
Neogene (Pliocene) 5.3–1.8	*Canoparmelia crozalsiana*-clade	3.91	1.68–7.09	3.36	1.77–5.36

Units. Ages: Ma. Rates: (s/s/y) ×10−9. HPD: highest posterior density interval.

The alternative analyses gave similar results (Table 3). The small differences are as follows:1) using two calibration points (C1 and C2) and excluding the *Alectoria* fossil, node ages and 95% HPD intervals are slightly younger for some of the parmelioid clades; 2) using C1 and C3 and excluding *Parmelia*, the node ages and 95% HPD intervals had a small decrease in older clades; and 3) using C1, C2 and the *Alectoria* fossil constraining the age of the alectorioid clade (*Alectoria*, *Bryoria* and *Pseudephebe*; C3*) the node ages are similar with very small fluctuations. The 95% HPD intervals for all the clades largely agree and are similar to those obtained in the first analysis using the calibration points C1, C2 and C3.

**Table 3:** Estimated ages of the most recent common ancestor (MRCA) of the main clades obtained in the alternative BEAST analyses.

	Only C1 + C2		Only C1 + C3		C1 + C2 + C3*	
Node MRCA	Age	Height 95% HPD	Age	Height 95% HPD	Age	Height 95% HPD
Lecanoromycetes	282.74	243.97–323.49	298.72	264.94–327.29	297.23	263.04–325.17
Ostropomycetidae	229.14	189.55–268.83	249.46	216.10–280.61	250.70	216.87–282.32
Lecanoromycetidae	225.43	182.87–266.59	240.92	196.18–278.16	239.00	199.59–275.59
Lecanorales	125.74	95.55–157.78	134.20	103.23–107.77	142.62	110.27–172.06
Parmeliaceae (*Protoparmelia* included)	88.03	64.84–114.27	91.88	67.9–118.82	111.24	83.53–137.09
Parmeliaceae (*Protoparmelia* excluded)	58.49	46.05–72.06	61.08	49.41–74.53	74.10	59.11–89.74
Parmelioid group	48.13	39.33–62.63	51.06	40.71–61.64	59.66	49.94–71.67
Split 1. *Xanthoparmelia* – *Parmotrema*-clade	38.77	29.41–49.12	41.86	30.78–52.92	48.27	38.52–59.90
Split 2. *Melanelixia* - *Melanohalea*	32.68	22.59–43.43	35.78	25.65–46.41	41.28	31.45–52.33
Split 3. *Parmelina* – *Myelochroa*	29.35	17.59–41.00	31.50	20.84–40.76	37.03	24.50–48.71
Split 4. *Remototrachyna* - *Bulbothrix*	27.29	20.11–37.92	27.56	20.59–36.92	32.91	23.80–42.46
Split 5. *Austroparmelina* - *Parmotrema* and *Flavoparmelia*	28.45	20.81–36.38	30.72	22.37–39.69	35.60	26.90–45.42
*Melanelixia*	26.67	18.49–35.80	29.49	19.90–39.12	33.56	24.14–44.57
Split 6. *Cetrariastrum*- *Hypotrachyna*-clade p.p.	26.34	19.07–35.42	27.14	20.79–35.27	31.31	22.72–40.53
Split 7. *Flavoparmelia* - *Parmotrema*	31.38	23.46–39.95	26.33	18.33–33.90	30.52	23.48–39.49
*Melanohalea*	19.62	11.10–29.61	21.23	13.64–31.13	25.41	16.94–37.06
*Parmelia*	21.98	17.01–28.37	11.59	5.42–20.11	23.63	17.53–31.92
*Remototrachyna*	19.42	13.21–29.44	19.74	13.46–28.16	23.52	16.67–32.68
*Flavoparmelia*	18.39	12.21–25.41	19.77	12.41–26.68	22.79	16.25–30.77
*Xanthoparmelia*	17.76	10.00–27.89	19.75	12.88–29.62	21.99	13.03–34.65
*Myelochroa*	13.04	7.66–19.72	14.25	8.15–21.12	15.69	9.39–23.09
*Punctelia*	11.00	6.82–15.87	12.15	10.62–24.19	14.18	9.89–21.29
*Parmotrema*	11.44	7.57–16.04	12.45	7.55–16.79	14.38	10.26–19.46
*Cetrelia*	7.68	3.38–14.17	8.19	3.23–15.00	9.28	4.34–18.38
*Parmelina*	6.08	1.89–12.77	6.96	2.18–14.96	7.90	3.20–16.03
*Canoparmelia crozalsiana*-clade	3.25	1.42–5.45	3.31	1.47–5.94	3.95	1.69–7.38

Using the following calibration points: (1) C1 and C2 only; (2) C1 and C3 only; and (3) C1, C2 and C3* (with *Alectoria* fossil assigned to the alectorioid crown).Units: Ma. HPD: highest posterior density interval.

The split of the Parmeliaceae core was estimated to be around 109 Ma (85.52–136.55 Ma) when the crustose genus *Protoparmelia* separated from the rest of the Parmeliaceae (Fig. 4, Table 2). The basal radiation of Parmeliaceae core took place between 60 and 74 Ma, when the main lineages of the family originated. The radiation of the large group of Parmelioid lichens was estimated to have begun in the Paleocene, around 60 Ma (49.81–73.55 Ma). The morphologically close but phylogenetically distant cetrarioid group, mostly distributed in temperate to alpine regions, was estimated to radiate in the Eocene (27 Ma, 18.81–37.90 Ma, Fig. 4) while the alectorioid clade radiated about 47 Ma (40.88–54.97 Ma).

Our analyses suggest seven separate major divergence events that led to the evolution of the main clades of parmelioid lichens (Fig. 4, marked with dots and numbers). The earliest divergence, estimated around 48 Ma, separated the *Xanthoparmelia*-clade from the *Parmotrema*-clade (including the genera *Austroparmelina*, *Canoparmelia*, *Flavoparmelia*, *Punctelia*, *Flavopunctelia*, *Nesolechia* and *Parmotrema*). In subsequent divergence events during Eocene time *Melanohalea* split from *Melanelixia* (about 42 Ma), *Parmelina* separated from *Myelochroa* (37 Ma), *Remototrachyna* from *Bulbothrix* (about 37 Ma) and *Austroparmelina* differentiated from the most recent common ancestor (MRCA) of *Parmotrema*, *Flavoparmelia* and *Canoparmelia crozalsiana*-clade (about 34 Ma). During the early Oligocene *Cetrariastrum* (about 32 Ma) differentiated from the rest of the complex *Hypotrachyna*-clade (including *Everniastrum*, *Hypotrachyna* s.l., and *Parmelinopsis*), and. *Flavoparmelia* separated from *Parmotrema* and *Canoparmelia crozalsiana*-clade (30 Ma).

Our data indicate that diversification of the recent genera of parmelioid lichens occurred during Oligocene and Miocene. The diversification of the *Melanelixia* was estimated to be around (34 Ma), and in a second radiation event (22–25 Ma) diversified *Melanohalea*, *Parmelia*, *Remototrachyna*, *Flavoparmelia* and *Xanthoparmelia*. *Myelochroa*, *Punctelia* and *Parmotrema* radiated during the Miocene (between 14 and 18 Ma), and *Cetrelia*, *Parmelina* and *Canoparmelia crozalsiana*-clade was estimated to radiated by late Miocene and early Pliocene (4–9 Ma).

**Figure 5:**
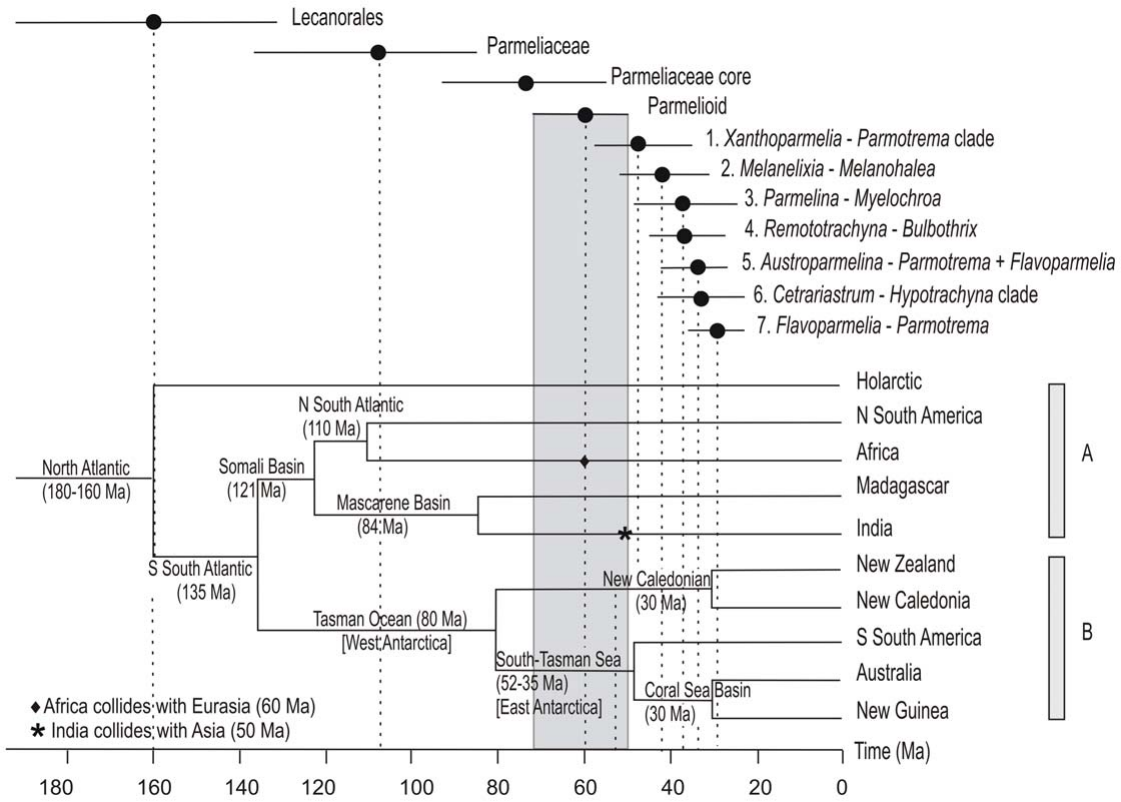
Comparison of divergence ages of parmelioid clades and the separation of the Southern Hemisphere landmasses. Dots represent node ages and bars cover the 95%HPD. The geological area cladogram representing the relationships among the Southern Hemisphere landmasses is based on Sanmartín & Ronquist [106].

The estimated ages of diversification of the main clades of parmelioid lichens compared to the geological area cladogram representing the relationships among the Southern Hemisphere landmasses are shown in Figure 5. The break-up of the main landmasses of the South Hemisphere predate the ages of diversification of the parmelioid clades and the splits of the main lineages (Fig. 5). The presence of several genera (e.g. *Xanthoparmelia*, *Parmotrema*, *Bulbothrix*, *Austroparmelina*, *Flavoparmelia*, *Melanelixia*, *Myelochroa*, *Hypotrachyna*, *Punctelia*) in different continents of the Southern Hemisphere (A and B, Fig. 5) cannot be explained by continental drift and vicariance because when the parmelioid lineages started to diverge (60 Ma) Africa had already separated from South America, India, New Zealand and Australia.

## Discussion

In this study we put a time-scale on the phylogenetic tree of parmelioid lichens using three DNA regions with different evolutionary rates. The estimated ages allow addressing how vicariance and long-distance dispersal shaped the current distribution patterns of parmelioid lichens, specially the disjunct distributions of species groups in the Southern Hemisphere. Moreover the dated phylogeny provides a general picture of the palaeoclimatic conditions prevalent on Earth when the main lineages differentiated.

The three DNA regions used in this study to build the phylogenetic trees have different evolutionary rates. We found higher substitution rates in the protein coding gene *RPB1* than in the nuLSU and mtSSU ribosomal DNA (Fig. 2, Table 2). Ribosomal DNA has been frequently used in molecular studies of Parmeliaceae and other lichenized fungi [34], [41]–[42], [44]–[47], [52]–[54] but so far few molecular studies have used protein-coding genes to infer phylogenetic relationships in Parmeliaceae [18], [35], [43], [55]–[56]. In our analysis the *RPB1* gene (with high substitution rates) provided better resolution of the terminal lineages of the tree while the more conserved genes with lower substitution rate (nuLSU, mtSSU) better supported the backbone of the tree topology.

Substitution rates have been used to estimate divergence times due to the lack of fossil records. Takamatsu & Matsuda [57] calculated a substitution rates for Erysiphales (2.52×10^−9^ s/s/y for nuLSU). Our estimation of the substitution rate for the nuLSU of the Parmeliaceae (1.12×10^−9^ s/s/y; Table 2) is in the same order of magnitude but slower than in the phytopathogenous fungi of Erysiphales. This indicates that caution should be used when applying substitution rates of a group of fungi to estimate divergence times of an unrelated group [58]–[59].

The scarcity and uncertainty of the fossil record was a major obstacle for estimation of dates of radiation in most groups of fungi. We have estimated divergence times of the major lineages of Parmeliaceae and times of the main radiations of the genera using a comprehensive phylogenetic hypothesis of the family, calibrated with the available fossil evidence, a root time inferred from [26], and allowing a relaxed-clock model for the rates of evolution of the main clades.

Berbee & Taylor [32] estimated the age of the parmeliaceae crown node at about 60 Ma based on substitution rates, and a minimum age of the family was given at about 40 Ma according to fossil records [50]–[51]. However, the age estimates obtained herein suggested that Parmeliaceae evolved much earlier. Our analyses indicate that the Parmeliaceae core originated rather recently with a stem node age estimate of 108 Ma (Fig. 3, 4, Table 2) and a crown node age estimate of 74 Ma (Fig. 4, Table 2). For other major families of lichenized ascomycetes much older crown node ages have been estimated (Rivas Plata & Lumbsch, pers. com.), including Graphidaceae (156 Ma), Physciaceae (153 Ma), and Ramalinaceae (126 Ma). The parmelioid clade is the largest and most strongly supported monophyletic group of the family. Within the parmelioid crown a total of seven major divergence events at different times have been found (Fig. 4), ranging from early Eocene (separation of *Xanthoparmelia* from the *Parmotrema*-clade) to Oligocene (separation of *Flavoparmelia* from *Parmotrema* and *Canoparmelia crozalsiana*-clade). The radiations of parmelioid genera were estimated to start at the end of Eocene (*Melanelixia*) and occurred during the Oligocene-Miocene for most of the genera.

Major clades of parmelioid lichens either show distinct distribution patterns of the clade or include numerous species with disjunct distributions, as in the genera *Xanthoparmelia*, *Austroparmelina*, *Melanohalea*, *Parmelina*, and *Remototrachyna* [11], [17], [19], [41], [60]–[64]. Examples of disjunct distributions include *Xanthoparmelia* and *Austroparmelina*. *Xanthoparmelia* with ca. 800 species, the most speciose clade of Parmeliaceae, occurs worldwide, although in some cases the species delimitation has recently been challenged [65], and in other cases they have been shown much more restricted than previously known [61]–[64]. Despite being cosmopolitan, *Xanthoparmelia* has two main areas of distribution in arid regions of the Southern Hemisphere (Australia and Africa). A distribution pattern spanning over Australia and Africa cannot be explained by vicariance since these landmasses separated much earlier (Fig. 5; 110–135 Ma) than the split of the *Xanthoparmelia* lineage from the *Parmotrema*-clade (37–58 Ma). The data, however, cannot reject vicariance as the reason for distribution ranges of species occurring in South America and Australia. The separation of these continents (35–52 Ma) happened at a similar time as the origin of this lineage.

*Austroparmelina* includes 13 species that occur in southern and eastern Australia, Tasmania and New Zealand. Two species have wider distribution areas, occurring also in South Africa (*A. labrosa*, *A. pseudorelicina*; [18]); and one species also is known from South America (*A. labrosa*, Chile; [66]). According to our estimates the genus separated from its sister lineages in the late Eocene. Thus the presence of *Austroparmelina* in South Africa is most plausibly explained as a result of long distance dispersal because separation of Africa took place much earlier (Fig. 5). As in the case of *Xanthoparmelia*, our data cannot reject vicariance events as an explanation for the presence of *A. labrosa* in South America and Australia.

In general the estimated ages of diversification events found in our analyses indicate that disjunct distribution patterns of Southern Hemisphere lineages cannot be explained by vicariance. Transoceanic long distance dispersal is the most plausible cause to explain these distribution patterns. This is especially true for taxa occurring in Africa and Australia. This is consistent with recent studies employing molecular clock approaches interpreting distribution patterns in other groups of fungi and plants [58]–[59], [67]–[76], suggesting that long-distance dispersal is an important factor to explain the current distribution patterns of plants and fungi. In the case of lineages distributed at present in South America, Antarctica and Australia the estimated ages do not discard that vicariance could have resulted from the break-up of continents that had occurred 35–52 Ma.

For genera with more restricted distribution ranges and species groups occurring mainly in the Holarctic, additional phylogeographical data are necessary to test biogeographical hypotheses. This is the case e.g., for *Parmelina*, a genus that occurs in areas with a Mediterranean climate in the Northern Hemisphere [11]. Its separation from *Myelochroa*, a genus with center of distribution in eastern Asia [77] but extends further into temperate regions, was estimated as having happened in the late Eocene. Similarly the recently described genus *Remototrachyna*, a Southeast Asian element with only one pantropical species [19], diverged from its sister-lineage *Bulbothrix* also in the late Eocene.

**Figure 6:**
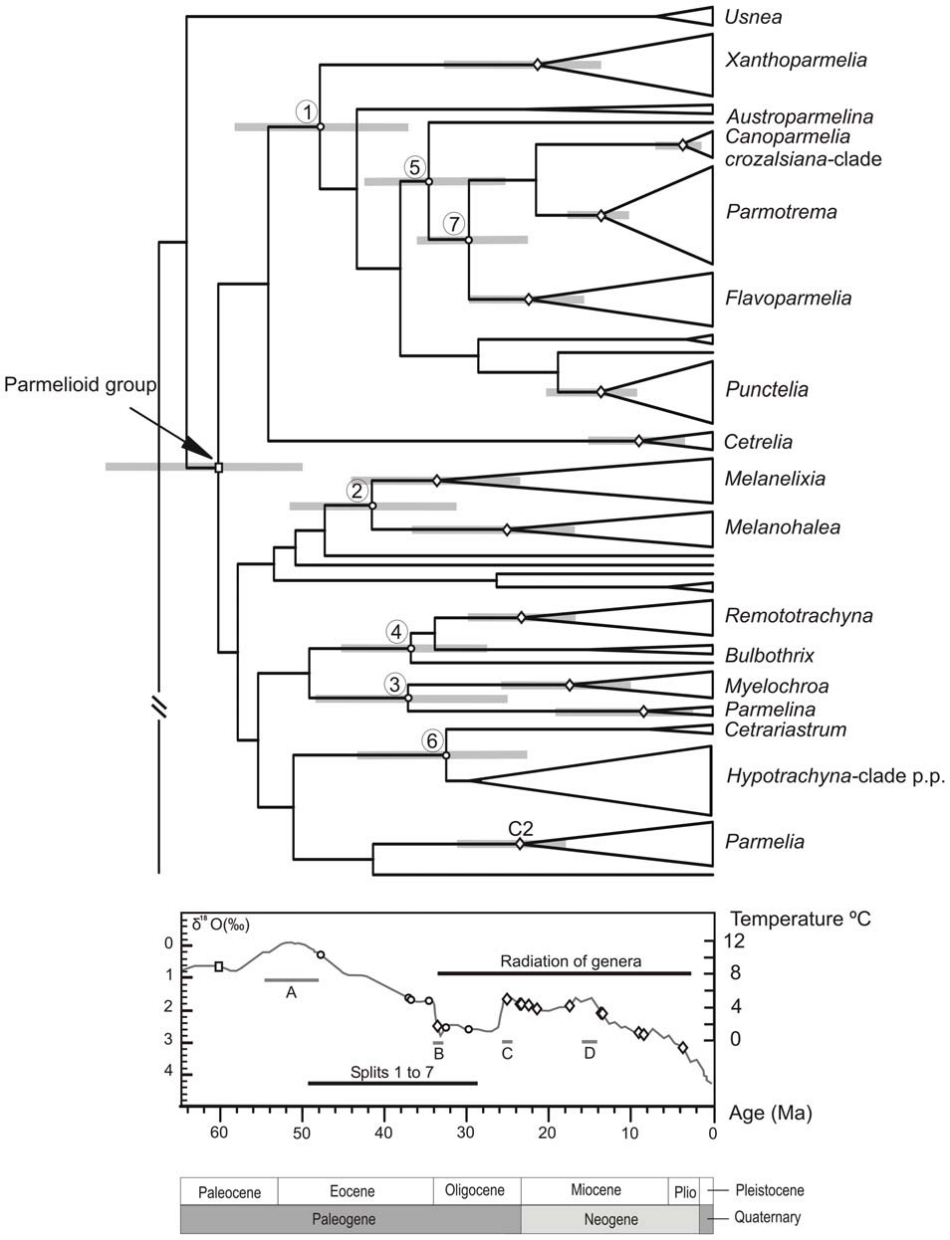
Chronogram of parmelioid genera during Cenozoic time and its relationship to global temperature changes. The global temperature changes obtained from the deep-sea oxygen and carbon isotype proxies after Zachos *et al* [107].The major diversification events of parmelioid lichens are mapped onto the temperature curve: the square represents the parmelioid most recent common ancestor (MCRA), circles indicate splits of major lineages (numbers in the tree refer to those in Table 2), and diamonds the radiation of genera. Climatic events: A. Early Eocene Climatic Optimum. B. Oi-1 Glaciation. C. Late Oligocene Warming. D. Mid-Miocene Climatic Optimum.

Our analyses suggest a complex relationship of diversification events and palaeoclimatic conditions. The origin of Parmeliaceae was estimated in the late Cretaceous when the climate was warmer than today, and when subtropical to temperate fauna and flora extended well into polar latitudes [78]–[79]. The first radiation of the parmelioid group (60 Ma) occurred just before the Early Eocene Climatic Optimum (52 Ma) (Fig. 6) when temperature and atmospheric CO_2_ reached maximum levels [80]. During this time period, the general climate was warm and humid, associated with tectonic changes and volcanism [81]–[82]. Most of the parmelioid lineages, however originated during the Eocene cooling and Oligocene glaciation (Fig. 6). During the Eocene–Oligocene transition (33.5 Ma) a profound global climate shift took place changing the Cretaceous/early Palaeogene “Green House” conditions to “Ice House” conditions, with the growth of Antarctic ice sheets to approximately their modern size, and the appearance of Northern Hemisphere glacial ice [80]. The radiation of the genera took place at different times from the early Oligocene to the Pliocene. *Melanelixia* started to radiate during the early Oligocene. During this time, the general climate was characterized for cold conditions of the Oligocene glaciation (B, Fig. 6). However *Melanohalea*, *Parmelia*, *Remototrachyna*, *Flavoparmelia*, *Xanthoparmelia* and *Myelochroa* started to radiate between the Late Oligocene Warming. and the Mid-Miocene Climatic Optimum (C–D, Fig. 6). The radiation of other genera (*Punctelia*, *Parmotrema*, *Cetrelia*, *Parmelina*, *Canoparmelia crozalsiana*-clade) is estimated as having occurred at the transition from the middle Miocene to the Early Pliocene, before the climate became cooler, drier and seasonal at the end of Pliocene [80].

### Conclusions

Using three calibration points (one at the split of Lecanoromycetes and Eurotiomycetes, and the ages of two fossil lichens) we obtained the first dated phylogeny of parmelioid lichens and estimated the ages of divergence of the well-resolved lineages and main genera. The radiation of the Parmelioid occurred near the Cretaceous-Tertiary (K/T) boundary, before the climatic optima. These age estimates indicate that long-distance dispersal has played a major role in shaping the current distribution of the Southern Hemisphere parmelioid lichens and that continental drift of Gondwana landmasses and vicariance cannot explain the Africa-Australia disjunct patterns. The major genera originated during Eocene and Oligocene, and radiated during cooling periods at different times from the late Oligocene to early Pliocene.

## Materials and Methods

### Taxon sampling, sequence alignment and selection of substitution model

A dataset of 225 specimens of Lecanoromycetes with complete sequences of nuLSU rDNA, mtSSU rDNA and the protein coding *RPB1* gene, generated in previous studies [18], [83]–[84], was compiled for this study. GenBank accession numbers are listed in Table S1. The parmelioid lineages (the main focus of this work) were represented by 96 OTUs including 24 genera. The major lineages of Lecanoromycetes [85]–[86] and outgroups (Chaethothyriomycetidae and Dothideomycetes), represented by 129 OTUs were included in the analyses to prevent that uneven sampling across the tree could distort the apparent trend in speciation through time [87]. The sequences of each locus were aligned separately using Muscle 3.6 [88] and the ambiguous positions removed using Gblocks with default settings [89]–[90]. The general time reversible model including estimation of invariant sites (GTR+I+G) was selected by jModelTest v 0.1.1 [91]–[92] as the most appropriate nucleotide substitution model for the three separated loci.

### Calibration of nodes and dating analysis

The divergence time analyses were performed using BEAST v.1.6.1 [93]. For the dating analysis it is recommended to use a user starting tree instead of the random starting tree built by BEAST. The latter is very likely to violate the temporal and/or topological constraints specified to calibrate divergence times, and cause an error when attempting to initiate the MCMC. For building this tree we checked the phylogenetic signal of our matrix running preliminary ML and Bayesian analyses using Garli 0.96 [94] and MrBayes 3.1.1 [95]. ML analyses were carried out with the default settings and Bayesian analyses were performed assuming a GTR+I+G model, run for 5 million of generations with 4 chains and every 100^th^ tree sampled. The first 5000 generations were burned in and a majority rule consensus tree was calculated with the sumt option. The topologies generated separately for each locus by ML and Bayesian analyses were congruent with the topology of the three loci concatenated, and with the general phylogeny obtained by Crespo *et al*. [18].

Three points of calibration were used for this study. The principal calibration point (C1) was the divergence time of 280–330 Ma for the stem of Lecanoromycetes following [26]. In addition we used the ages of two fossil lichens: the diversification node (C2) of *Parmelia* was calibrated with fossils from the Dominican amber (*Parmelia ambra*, 15–45 Ma, [50]), and the crown node (C3) of *Alectoria* with a fossil from the Baltic amber (35–40 Ma, [49]). The assignation of fossils to extant groups is a crucial matter in dating analyses, and in the case of the lichens the fossil record is so sparse that this becomes particularly important. *Parmelia ambra* is a fossil from the Dominican amber resembling *Parmelia saxatilis* and similar species [50]. It presents unclear terminal pseudocyphellae, elongate isidia, plane to concave upper surface, and simple to dichotomously branched rhizines. All these features are characteristic of the *Parmelia s. s.* clade, and thus the fossil could also be related to the phylogenetically close genus *Relicina* (sister group of the *Parmelia* s.s.), the ‘*Parmelia signifera*’ group, or to the morphologically close *Nipponoparmelia* [18]. *Relicina* was discarded because *P. ambra* does not have cilia, a feature present on the *Relicina* species. The species of the ‘*Parmelia signifera*’ group have subsquarrose rhizines different from the simple to dichotomously branched rhizines of the fossil. On the other hand, *Nipponoparmelia* presents lobes rolled upwards [96] different from the flat lobes of the fossil. Moreover, only *N. isidioclada* has isidia but the rhizines are much branched, not simple or bifurcate, than those of the fossil. Thus, the *Parmelia ambra* fossil was used to calibrate the *Parmelia* s. s. crown node in the starting tree.

The *Alectoria* fossil from the Baltic amber [49] is morphologically related to the alectorioid clade (*Bryoria*, *Pseudophebe* and *Alectoria*; [35]). The fossil has abundant apothecia, a character that it shares with *Alectoria*, while the related *Bryoria* and *Pseudephebe* genera rarely have apothecia. Thus this fossil was used to constrain the crown node of *Alectoria*. Nevertheless, due to the inevitable uncertainty in placement of fossil taxa we assessed the impact of individual fossil calibration on divergence time estimates using alternative analysis: 1) using a single fossil for calibration, either *Parmelia* (C2 node) or *Alectoria* (C3 node); and 2) using the *Alectoria* fossil to calibrate the whole alectorioid clade, including *Alectoria*, *Bryoria* and *Pseudephebe* (C3*).

The divergence time analyses were performed using BEAST v.1.6.1 [93]. We used as starting tree the ML tree obtained with Garli 0.96 [94], made ultrametric using nonparametric rate smoothing (NPRS) implemented in TreeEdit v.10a10 [97] with the divergence between Lecanoromycetes and Chaethothyriomycetidae set at 305 Ma. We constrained the position of Chaethothyriomycetidae as sister clade of Lecanoromycetes based on Schoch *et al*. [98]. Previously to the analysis, we test that this constraint is not significantly worse than the unconstrained topology, using the Shimodaira-Hasegawa test (SH) [99] and Expected Likelihood Weights test (ELW) [100]. Both tests were run on Tree-Puzzle 5.2 [101].

The final dating analysis was performed with a partitioned BEAST analysis with unlinked substitutions models (GTR+I+G) across the loci, a Birth-Death process tree prior, and a relaxed clock model (uncorrelated lognormal) for each partition. Calibration points were defined as prior distributions: 1) the split of Lecanoromycetes and Chaethothyriomycetidae (C1) was calibrated with a uniform distribution (280–330 Ma). 2) The calibrations points with fossils were considered as minimal ages and calibrated with a lognormal distribution [102]. The *Parmelia* crown node (C2) at log-normal mean = 2.77, offset = 14, lognormal standard deviation = 0.5. The *Alectoria* crown node (C3) at lognormal mean = 3.61, offset = 34, lognormal standard deviation = 0.75. The final analysis was run for 10 million generations, with parameter values sampled every 1000 generation. We checked the stationary plateau with Tracer v. 1.4.1 [103]. We discarded 10% of the initial trees as burn in and the consensus tree was calculated using Tree Annotator v 1.6.1 [96]. The results were visualized with FigTree v. 1.3.1 [104]. The substitution rates for each locus were obtained running independent BEAST analyses for each dataset using the same parameters as in the partitioned analysis. Ages and rates were estimated for all the nodes with more than 0.95 of posterior probability both in the BEAST runs and in the previous Bayesian analysis.

## Supporting Information

Table S1 **Specimens used in this study with GenBank accession numbers.**
(DOC)
