# Supplementary material for: Origin and Diversification of Major Clades in Parmelioid Lichens (Parmeliaceae, Ascomycota) during the Paleogene Inferred by Bayesian Analysis
Source: PLoS One. 2011 Dec 8;6(12):e28161. doi: 10.1371/journal.pone.0028161 (PMC3234259; doi:10.1371/journal.pone.0028161)
Supplement: Table S1 — Specimens used in this study with GenBank accession numbers. (DOC) [file pone.0028161.s001.doc]

|  | GenBank accession no. |  |  |
| --- | --- | --- | --- |
| Species | nuLSU | mtSSU | *rpb1* |
| *Acarospora schleicheri* | AY853353 | AY853305 | DQ915591 |
| *Ainoa geochroa* | DQ871006 | DQ871015 | DQ870927 |
| *Ainoa mooreana* | AY212828 | AY212850 | DQ870928 |
| *Alectoria nigricans* | DQ923649 | DQ923620 | DQ923676 |
| *Alectoria ochroleuca* | DQ899288 | DQ899289 | DQ923677 |
| *Alectoria sarmentosa* | DQ899290 | DQ899291 | DQ923678 |
| *Allantoparmelia alpicola* | DQ923650 | DQ923621 | DQ923679 |
| *Arctomia delicatula* | AY853355 | AY853307 | DQ870929 |
| *Arctomia teretiuscula* | DQ007346 | DQ007349 | DQ870930 |
| *Arthrorhaphis citrinella* | AY853356 | AY853308 | DQ915592 |
| *Aspicilia caesiocinerea* | DQ780303 | DQ780271 | DQ870931 |
| *Aspicilia cinerea* | DQ780304 | DQ780272 | DQ870932 |
| *Aspicilia izcoana* | AY853359 | AY853311 | DQ870934 |
| *Austroparmelina pruinata* | EF042914 | EF025481 | GU994680 |
| *Arctocetraria andrejevii* | DQ923652 | DQ923623 | DQ923680 |
| *Arctoparmelia centrifuga* | AY578917 | AF351156 | EF092099 |
| *Baeomyces placophyllus* | AY300658 | AF356878 | DQ870936 |
| *Baeomyces rufus* | DQ871008 | DQ871016 | DQ870937 |
| *Brodoa atrofusca* | AY607824 | AY643090 | EF092100 |
| *Brodoa intestiniformis* | DQ923653 | DQ923624 | DQ923681 |
| *Brodoa oroarctica* | DQ923654 | DQ923625 | DQ923682 |
| *Bryoria capillaris* | DQ923655 | DQ923626 | DQ923683 |
| *Bryoria fremontii* | DQ923656 | DQ923627 | DQ923684 |
| *Bryoria fuscescens* | EF042912 | AF351158 | EF092101 |
| *Bulbothrix coronata* | EU562671 | DQ287789 | GU994681 |
| *Bulbothrix decurtata* | EU562672 | DQ287790 | GU994682 |
| *Bulbothrix meizospora* | AY607780 | AY611127 | EF092102 |
| *Caloplaca flavorubescens* | AY300831 | AY143403 | DQ915593 |
| *Candelaria concolor* | EF436461 | EF436460 | EF436461 |
| *Candelariella aurella* | AY853361 | AY853313 | DQ915594 |
| *Cladonia rangiferina* | AY300832 | AY300881 | DQ915595 |
| *Cladosporium* sp*.* | AY016367 | AY571386 | DQ870938 |
| *Canoparmelia carneopruinata* | EF042913 | EF025480 | GU994684 |
| *Canoparmelia caroliniana* | AY584634 | AY584613 | DQ782817 |
| *Canoparmelia crozalsiana* | AY584831 | AY586594 | EF092104 |
| *Canoparmelia inhaminensis* | GU994586 | GU994633 | GU994686 |
| *Canoparmelia schelpei* | GU994588 | GU994634 | GU994688 |
| *Canoparmelia texana* | EF042915 | EF025482 | EF092105 |
| *Cetraria islandica 1* | DQ912334 | AY340486 | DQ912356 |
| *Cetraria islandica 2* | AY340539 | DQ912277 | DQ923685 |
| *Cetrariastrum andense* | GQ919245 | GQ919217 | GU994690 |
| *Cetrariastrum dubitans* | GQ919246 | GQ919217 | GU994691 |
| *Cetrariella delisei* | DQ923657 | DQ923628 | DQ923686. |
| *Cetrelia cetrarioides* | GU994591 | GU994636 | GU994692 |
| *Cetrelia olivetorum* | DQ923659 | DQ923630 | GU994693 |
| *Cetrelia pseudolivetorum* | GU994594 | GU994639 | GU994694 |
| *Coccotrema cucurbitula* | AF274092 | AF329161 | DQ870939 |
| *Coccotrema pocillarium* | AF274093 | AF329166 | DQ870940 |
| *Cornicularia normoerica* | DQ923661 | DQ923632 | DQ923687 |
| *Dactylina arctica* | DQ986802 | DQ986786 | DQ986859 |
| *Diploschistes cinereocaesius* | AY300835 | AY300885 | DQ870941 |
| *Diploschistes scruposus* | AF279389 | AY584692 | DQ870943 |
| *Emodomelanelia masonii* | GU994595 | GU994640 | GU994695 |
| *Evernia prunastri 1* | AF107562 | DQ923634 | EF105428 |
| *Evernia prunastri 2* | AF113745 | AF351162 | DQ870944 |
| *Everniastrum nepalense* | AY607783 | AY611129 | EF092106 |
| *Everniopsis trulla* | EF108290 | EF108289 | EF105429 |
| *Flavocetraria nivalis 1* | DQ883795 | DQ923635 | DQ883738 |
| *Flavocetraria nivalis 2* | AY533003 | DQ912278 | DQ923688 |
| *Flavoparmelia caperata 1* | AY584639 | AY584617 | EF092107 |
| *Flavoparmelia caperata 2* | AY578922 | AF351163 | DQ870945 |
| *Flavoparmelia citrinescens* | GU994596 | GU994641 | GU994696 |
| *Flavoparmelia marchantii* | GU994598 | GU994642 | GU994698 |
| *Flavoparmelia soredians* | AY584835 | AY586586 | EF092108 |
| *Flavoparmelia springtonensis* | EF042916 | EF025483 | EF092109 |
| *Flavoparmelia subambigua* | GU994599 | GU994643 | GU994699 |
| *Flavopunctelia flaventior 1* | DQ912335 | DQ912279 | EF092110 |
| *Flavopunctelia flaventior 2* | AY578923 | AY586587 | DQ912357 |
| *Graphis scripta* | AY853370 | AY853322 | DQ870947 |
| *Gregorella humida* | AY853378 | AY853329 | DQ870946 |
| *Hypocenomyce scalaris* | AY853373 | AY853325 | DQ915596 |
| *Hypogymnia vittata* | DQ900637 | DQ900629 | DQ923689 |
| *Hypotrachyna polydactyla* | GQ919258 | GQ919231 | GU994703 |
| *Hypotrachyna revoluta* | AY607787 | AF351166 | EF092112 |
| *Hypotrachyna sinuosa* | AY607788 | AY611133 | EF092113 |
| *Imshaugia aleurites 1* | DQ986753 | DQ986864 | EF092114 |
| *Imshaugia aleurites 2* | AY607840 | AF351167 | DQ986825 |
| *Lecanora hybocarpa* | EF105421 | EF105417 | DQ870949 |
| *Lecanora paramerae* | EF105422 | EF105418 | DQ870950 |
| *Lecanora sulphurea* | EF105423 | EF105419 | DQ870951 |
| *Lepraria usnica* | AY300843 | AY300894 | DQ870952 |
| *Lethariella cashmeriana* | DQ923665 | DQ923637 | DQ923690 |
| *Lobaria pulmonaria* | AF183934 | AF069541 | DQ915597 |
| *Lobothallia radiosa* | DQ780306 | DQ780274 | DQ870954 |
| *Loxospora ochrophaea* | DQ871009 | DQ871017 | DQ870953 |
| *Masonhalea richardsonii* | DQ973031 | DQ972979 | DQ973054 |
| *Melanelia disjuncta* | AJ421431 | DQ923638 | DQ923691 |
| *Melanelia hepatizon* | DQ923667 | DQ923639 | DQ923692. |
| *Melanelia sorediata* | GU994604 | GU994645 | GU994706 |
| *Melanelia sorediella* | GU994606 | GU994646 | GU994707 |
| *Melanelia stygia* | AJ421434 | DQ923640 | DQ923693 |
| *Melanelixia fuliginosa 1* | AJ421428 | DQ986787 | EF092116 |
| *Melanelixia fuliginosa 2* | AJ421435 | AY611179 | DQ986860 |
| *Melanelixia glabra* | AJ421427 | GU994651 | EF092118 |
| *Melanelixia subargentifera* | AJ421429 | AY611155 | EF092119 |
| *Melanelixia subaurifera* | AJ421432 | AY611174 | EF092120 |
| *Melanelixia subglabra* | GU994610 | GU994654 | GU994711 |
| *Melanohalea elegantula* | AJ421437 | AY611151 | EF092122 |
| *Melanohalea exasperata 1* | AJ421438 | AY611140 | EF092124 |
| *Melanohalea exasperata 2* | AY607795 | AY611138 | EF092123 |
| *Melanohalea olivacea* | AY607803 | AY611148 | EF092125 |
| *Melanohalea subelegantula* | AY607829 | AY611171 | EF092126 |
| *Menegazzia terebrata* | AY584637 | DQ899305 | DQ923694 |
| *Myelochroa aurulenta 1* | DQ973025 | EF025484 | DQ973049 |
| *Myelochroa aurulenta 2* | EF042917 | DQ972972 | EF092127 |
| *Myelochroa irrugans* | AY607815 | AY611160 | EF092128 |
| *Myelochroa metarevoluta* | AY607814 | AY611159 | EF092129 |
| *Myriangium duriaei* | AY016365 | AY571389 | DQ870956 |
| *Nesolechia oxyspora* | DQ923669 | DQ923642 | GU994712 |
| *Ochrolechia androgyna* | AY300846 | AY300897 | DQ870957 |
| *Ochrolechia oregonensis* | DQ780308 | DQ780276 | DQ870958 |
| *Ochrolechia parella* | AF274097 | AF320173 | DQ870959 |
| *Ochrolechia turneri* | AY568002 | AY567982 | DQ870961 |
| *Omphalodium pisacomense* | GU994617 | GU994663 | GU994715 |
| *Orceolina antarctica* | AY212115 | AF274852 | DQ870962 |
| *Orceolina kerguelensis* | AY212830 | AF381561 | DQ870963 |
| *Pannoparmelia angustata* | AY785265 | AF351170 | EF092131 |
| *Parmelia discordans* | EF042918 | DQ287841 | EF092132 |
| *Parmelia saxatilis* | AY300849 | AY340514 | DQ923695 |
| *Parmelia serrana* | AY578948 | AY582319 | EF092133 |
| *Parmelia squarrosa* | AY607816 | AY611162 | EF092134 |
| *Parmelia sulcata* | AY578949 | AY582320 | EF092135 |
| *Parmelia sulcata 2* | GU994669 | GU994621 | GU994720 |
| *Parmelina carporrhizans* | AY607818 | AY611164 | EF092136 |
| *Parmelina tiliacea* | AY578950 | AF351173 | EF092137 |
| *Parmelinopsis afrorevoluta* | GQ919259 | GQ919233 | GU994722 |
| *Parmelinopsis horrescens* | AY578951 | AY582321 | EF092138 |
| *Parmelinopsis minarum* | AY578952 | AY582322 | EF092139 |
| *Parmelinopsis neodamaziana* | AY607820 | AY611166 | EF092140 |
| *Parmelinopsis subfatiscens* | AY607821 | AF351174 | EF092141 |
| *Parmeliopsis hyperopta* | AY607823 | AY611167 | EF092142 |
| *Parmotrema cetratum* | AY584847 | AY648935 | EF092143 |
| *Parmotrema crinitum* | AY584837 | EU562699 | GU994723 |
| *Parmotrema fistulatum* | AY578920 | EU562700 | GU994724 |
| *Parmotrema haitiense* | AY578918 | AY582295 | EF092144 |
| *Parmotrema hypoleucinum* | AY584839 | AY586590 | GU994725 |
| *Parmotrema perforatum* | AY584840 | AY586591 | EF092145 |
| *Parmotrema perlatum* | AY584838 | AY586580 | EF092146 |
| *Parmotrema pilosum* | AY578919 | EU562701 | GU994728 |
| *Parmotrema reticulatum 1* | DQ912339 | DQ912283 | DQ912361 |
| *Parmotrema reticulatum 2* | AY584850 | AY648933 | GU994729 |
| *Parmotrema subtinctorium* | AY584830 | AY586582 | GU994730 |
| *Parmotrema tinctorum* | AY584635 | AY584627 | DQ912362 |
| *Peltigera aphthosa* | AF286759 | AY340515 | DQ915598 |
| *Pertusaria albescens* | AF329176 | AF329175 | DQ870964 |
| *Pertusaria amara* | AF274101 | AY300900 | DQ870965 |
| *Pertusaria coccodes* | AF279295 | AY567984 | DQ870966 |
| *Pertusaria corallophora* | DQ780316 | DQ780285 | DQ870969 |
| *Pertusaria coronata* | AY300851 | AY300902 | DQ879068 |
| *Pertusaria gibberosa* | DQ780322 | DQ780289 | DQ870970 |
| *Pertusaria hemisphaerica* | AF381556 | AF381563 | DQ902341 |
| *Pertusaria lactea* | AF381557 | AF381564 | DQ870971 |
| *Pertusaria lecanina* | AF279296 | AY567991 | DQ870972 |
| *Pertusaria leioplaca* | AY300852 | AY300903 | DQ870973 |
| *Pertusaria mammosa* | AY212831 | AY212854 | DQ870974 |
| *Pertusaria mesotrapa* | DQ780325 | DQ780292 | DQ870975 |
| *Pertusaria ophthalmiza* | AY568006 | AY567993 | DQ870976 |
| *Pertusaria panyrga* | DQ780327 | AY567994 | DQ870977 |
| *Pertusaria pertusa* | AF279300 | AF381565 | DQ870978 |
| *Pertusaria plittiana* | DQ780328 | DQ780294 | DQ870979 |
| *Pertusaria scaberula* | AF274099 | AF431959 | DQ870980 |
| *Pertusaria subventosa* | AY300854 | AY300905 | DQ870981 |
| *Pertusaria velata* | AY300855 | AY300906 | DQ870982 |
| *Placopsis cribellans* | DQ871010 | DQ871018 | DQ870983 |
| *Placopsis gelida* | AY212836 | AY212859 | DQ870984 |
| *Placopsis santessonii* | AY212845 | AY212867 | DQ870986 |
| *Placynthiella icmalea* | AY212846 | AY212870 | DQ870985 |
| *Platismatia glauca 1* | DQ973032 | AY756404 | DQ912363 |
| *Platismatia glauca 2* | DQ912340 | DQ972980 | DQ973055 |
| *Platismatia norvegica* | DQ923671 | DQ923644 | DQ923696 |
| *Pleurosticta acetabulum* | AY578953 | AY582323 | EF092147 |
| *Protoparmelia badia 1* | DQ431917 | EF105420 | EF105434 |
| *Protoparmelia badia 2* | DQ431916 | DQ899311 | DQ870987 |
| *Protothelenella corrosa* | AY607734 | AY607746 | DQ870988 |
| *Protothelenella sphinctrinoidella* | AY607735 | AY607747 | DQ870989 |
| *Protousnea magellanica* | DQ985193 | DQ985194 | DQ985195 |
| *Pseudephebe pubescens* | AY607839 | AF351180 | EF092148 |
| *Pseudevernia consocians* | DQ986754 | DQ986868 | DQ986826 |
| *Pseudevernia furfuracea* | AY607826 | AF351181 | EF105435 |
| *Punctelia borreri* | AY578954 | AY582324 | EF092149 |
| *Punctelia jeckeri* | AY613427 | GU994625 | GU994731 |
| *Punctelia pseudocoralloidea* | AY584843 | AY586595 | EF092150 |
| *Punctelia reddenda* | GU994627 | AY613430 | GU994732 |
| *Punctelia rudecta 1* | AY584636 | AY584630 | EF092151 |
| *Punctelia rudecta 2* | AY584845 | GU994672 | DQ912365 |
| *Punctelia* sp*.* | GU994628 | GU994673 | GU994733 |
| *Punctelia subflava* | AY584846 | EU562704 | GU994734 |
| *Relicina subnigra* | AY785267 | AY785281 | EF092152 |
| *Remototrachyna ciliata* | AY785266 | AY785280 | EF092111 |
| *Remototrachyna costaricensis* | AY785262 | AY785276 | GU994736 |
| *Remototrachyna incognita* | EU562687 | DQ287815 | GU994737 |
| *Remototrachyna infirma* | AY785264 | AY785278 | GU994738 |
| *Remototrachyna scytophylla* | EU562694 | DQ287836 | GU994739 |
| *Rhizocarpon sphaerosporum* | AY853390 | AY853340 | DQ870991 |
| *Rimularia psephota* | DQ871012 | DQ871019 | DQ870992 |
| *Schaereria corticola* | AY300859 | AY300909 | DQ870993 |
| *Sporastatia testudinea* | AY640969 | AY584725 | DQ870994 |
| *Staurothele fissa* | DQ329028 | DQ329003 | DQ870995 |
| *Staurothele rufa* | DQ329029 | DQ329004 | DQ70996 |
| *Thamnolia vermicularis* | AY853395 | AY853345 | DQ915599 |
| *Thelotrema subtile* | DQ871013 | DQ871020 | DQ870997 |
| *Thelotrema suecicum* | AY300867 | AY300917 | DQ870998 |
| *Trapelia chiodectonoides* | AY212847 | AY2128873 | DQ870999 |
| *Trapelia placodioides* | AF274103 | AF431962 | DQ366259 |
| *Trapeliopsis flexuosa* | AF274118 | AY212875 | DQ871000 |
| *Trapeliopsis granulosa* | AF274119 | AF381561 | DQ871001 |
| *Trapeliopsis percrenata* | AF279302 | AY212876 | EF158853 |
| *Tuckermannopsis chlorophylla* | DQ923674 | DQ923647 | DQ923697 |
| *Umbilicaria crustulosa* | AY300869 | AY300919 | DQ871002 |
| *Umbilicaria decussata* | AY603113 | DQ571021 | DQ871003 |
| *Umbilicaria hyperborea* | AY853399 | AY853349 | DQ915600 |
| *Usnea antarctica 1* | DQ883692 | DQ990920 | DQ883721 |
| *Usnea antarctica 2* | DQ899309 | EF116571 | EF193050 |
| *Usnea trachycarpa* | EF116570 | EF116572 | EF193058 |
| *Vulpicida pinastri 1* | DQ912341 | DQ923648 | DQ912366 |
| *Vulpicida pinastri 2* | DQ923675 | DQ912285 | DQ923698 |
| *Wawea fruticulosa* | DQ007347 | DQ871023 | DQ871005 |
| *Xanthoparmelia azaniensis* | EF042910 | EF025478 | EF092098 |
| *Xanthoparmelia conspersa 1* | AY584641 | DQ899314 | EF092155 |
| *Xanthoparmelia conspersa 2* | DQ899313 | AY584633 | DQ912367 |
| *Xanthoparmelia exornata* | EF108318 | EF025485 | EF092130 |
| *Xanthoparmelia hottentotta* | EF042919 | EF025486 | EF092153 |
| *Xanthoparmelia mougeotii* | AY578966 | AY582336 | EF092156 |
| *Xanthoparmelia saxeti* | AY578926 | AY582299 | EF092115 |
| *Xanthoparmelia semiviridis* | AY578921 | AF351160 | EF092157 |
